# Screening for developmental delay among children attending a rural community welfare clinic in Ghana

**DOI:** 10.1186/1471-2431-13-119

**Published:** 2013-08-13

**Authors:** Ajediran I Bello, Jonathan NA Quartey, Louisa A Appiah

**Affiliations:** 1 Department of Physiotherapy, School of Allied Health Sciences, College of Health Sciences, University of Ghana, Accra, Ghana

## Abstract

**Background:**

Periodic screening for developmental delays (DD) could avert the incidence of disability among children. However, such routine programme is yet to take off in rural welfare clinics in Ghana.

**Method:**

Mothers of under-5 children who were attending rural child welfare clinic participated in this study. The socio-demographic data of the mothers and their children were recorded. The children were screened to assess their gross motor skills, fine motor skills, communication skills, problem solving/cognition and social/personal interaction using Ages and Stages Questionnaire. Score below the threshold points on a developmental domain defines DD for a child. Data analysis involved percentages and frequency while Chi-square was performed to determine the associations between the selected socio-demographic risk factors and DD. Alpha value was set at p < 0.05.

**Results:**

Three hundren and thirty (330) children were screened and majority 60(18%), were found within the age range 3 months 1 day to 5 months 0 day. 251(76%) had normal weight (2.5 kg-3.5) while 26(7.6%) were underweight (<2.5 kg). Generally, 147(44.6%) of the children had DD in the different domains of the questionnaires. 41(12.4%) had DD in social/personal interaction while 19(5.8%) were delayed in the communication domain. Birth weight and duration of gestation were significantly associated with communication domain while the level of education of the mothers and duration of gestation were significantly associated with gross motor domain.

**Conclusion:**

An appreciable proportion of the children were found to experience developmental delays and the most prevalent occurence was in personal/social interaction. Birth weight, gestational age and maternal educational level provide insight into a link with communication and gross motor skills.

## Background

Child health and development form part of the core components of the millenium development goals set by the United Nations Member States to be achieved by the year 2015. However, this laudable goal can only be fully achieved with due consideration of health surveilliance of pre-school age children. Developmental delay (DD) occurs when a child does not reach developmental milestones at the expected age [1]. Five key domains of development abound for children under five years of age namely; gross motor skills, fine motor skills, communication skills, cognition skills and social/personal activities [2]. These are expected changes in skill developments that a child must pass through at predictable periods and in a predictable manner [3].

A child may be affected in one or more of these domains during growth and development which underscores the importance of proper developmental screening [4]. Prevention of disabilities in infants is often beset with problems including non-detection or late identification of delayed development. A survey conducted in the United State of America revealed that, about 16% of children are affected by various disabilities caused by speech and language delay, mental retardation, learning disabilities and emotional/behavioural problems, however only 30% of such children were identified before school entrance age [5]. According to the World Health Organization (WHO), about 5% of the world’s children who were below 14 years of age suffered from moderate to severe DD-associated disability most of which would have been either prevented or managed, if detected early [6]. Another study also found that 8% of all pre-school children from birth to 6 years had developmental problems and showed delay in one or more developmental domains [7]. Similarly, a related report from Central Region of Ghana indicated that 1.8% of disabilities were found among 2556 sampled Children who were less than 15 years [8]. These findings raise questions about the timelines at which these disabilities were detected and by what approach.

In the developed countries, there is a general concensus regarding the importance of monitoring children’s development through systematic screening [9]. Developmental screening is a globally adopted measure by which children at various set ages (2 to 60 months) are routinely assessed to detect those at high risk for significant unsuspected deviation from normal. The screening forms part of the key components in preventive care of children with a view to facilitate early identification and referral of the affected infants and children who need early intervention [10-12].

Based on anecdotal observation, developmental screening for children from birth to five years of age is not commonly practiced as part of the services rendered at rural child welfare clinics in Ghana. This shortcoming is speculated to be the reason why children with disabilites are often detected late when dysfunctions or inefficient movement behaviour have already emerged [13]. Moreover, identification of DD is exclusively done by health care professionals with little or no involvement of parents or caregivers. Meanwhile, parents’ descriptions of children’s abilities have been reported to be generally reliable with correct suspicion of their children’s probable developmental abnormality [14]. In a study of pre-school aged children referred for comprehensive paediatrics assessment, parents’ developmental concerns were confirmed for more than 90% of the children [15]. This suggests a profound interest of parents in their children’s growth and development.

Given the shortage of health care personnel in most rural communities in which just a quarter of the nation’s health care facilities are located, introduction of parent-centered developmental screening of children under the age of five years may pave way for early detection of DD with a view to contribute to the overall health care needs of children in Ghana. This study therefore sought to screen the children under five years of age for developmental delay in a rural Welfare Clinic with special consideration to their socio-demographic risk factors.

## Methods

### Participants

Participants for this cross-sectional study were mothers of children whose ages were less than 5 years and who were attending the out-patient department of a rural welfare clinic in Ghana. They were recruited through sample of convenience method. Participants were included if they had profound understanding of Twi language (a widely spoken local language in Ghana), their children had not been diagnosed of any neuro-developmental problems and had no febrile illnesses that could affect their required performances on a specific developmental domain at the time of screening. Mothers whose children were older than 60 months were excluded. The sample size for the participants was determined using the formula: N = Z^2^ (pq)/e^2^[16]. Where, N = Minimum sample size

Z = z-value at α = 0.05 = 1.96

p = 50% chance of occurrence of developmental delay = 0.5

q = 50% chance of non-occurrence of developmental delay = 0.5

e = allowable error = 0.05

Thus, the minimum sample size (N) proposed for this study = 1.96^2^ (0.5) (0.5)/(0.05)^2^ = 384.

### Survey instrument

The Ages and Stages Questionnaire was used to screen the children in this study [17]. It is composed of 21 sets of questionnaires covering age range 2 to 60 months. For instance, the 2 month questionnaire is defined as 1 month 0 days through 2 months 30 days. The questionnaire covers the five key developmental areas namely; gross motor skills, fine motor skills, communication skills, problem solving/cognition skills and social/personal interaction. Each set is composed of 30 items; 6 in each domain. Responses to items in all the domains are scored as follows: “yes” response (10 points), “sometimes” response (5 points) and “not yet” response (0 points). The maximum score in each domain is 60 points. In any set of the questionnaire, a child must be referred for further assessment if his/her scores fall short of a given cut-off point for any developmental domain. The tool is applicable as researcher-administered and self-administered assessment form.

### Procedure

The study was approved by the Ethical and Protocol Review Committee of the University of Ghana Medical School. Written informed consent was obtained from each participant following explicit information regarding the research procedure through signing or thumb printing. On enrollment, detailed history including demographic (the maternal and child age, educational qualification of the mother/caregivers and occupation) and medical/social (parity, duration of gestation and birth weight of the children) variables were collected and recorded. The Ages and Stages Questionnaires were administered to all the eligible mothers who consented to participate in the study according to the ages of their children. The items on the questionnaires were explained to the participants where necessary. Mothers/caregivers who could not readily respond to some items on the first visit were required to observe and respond at their next visit. The choice of this instrument is informed by its liberal mode of administration.

### Data analysis

Data were analyzed using Statistical Package for Social Science (SPSS) software package version 19. Summary of data were presented using simple percentage and frequency. The associations between developmental delay and the selected risk factors were determined using Chi square. Level of significance was set at 0.05.

## Results

### Socio-demographic characteristics of the sampled mothers and their children

**Figure 1:**
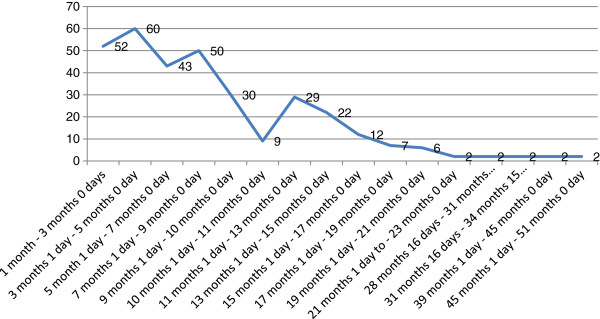
Age distribution of the sampled children.

A total of three hundred and eight-nine (389) children were screened in this study out of which data for three hundred thirty (330) were valid for analysis accounting for 85% response rate. Details of socio-demographic characteristics of the children are shown in Table 1. One hundred and seventy-three (52.4%) of the children screened were males. Two Hundred and fifty one (76%) had normal body weight (2.5 kg-3.5) as against 26(7.6%) who had low body weight (<2.5 kg). One hundred and sixty-two (49.7%) of the children were delivered through spontaneous vertex delivery and 63(19.1%) of them were delivered before term. The age category of the screened children showed that majority, 60(18%) were found within the age range 3 months 1 day to 5 months 0 day (Figure 1). Maternal age at delivery was within the age range 25-29 years for 123(37.3%) participants and very few, 2(0.6%) were found in the age range 45-49 years. Also, 133(40.3%) of the participants attained Junior High School level of education while 106(32.1%) had 2 children each (Table 2). A total of 147(44.6%) of the children had DD in various domains of the Ages and Stages Questionnaires. Forty-one, (12.4%) of the children had DD in social/personal interraction and 48(14.1%) of them were at risk of being delayed. In addition, 19(5.8%) of them were delayed in communication domain while 30(9.1%) were at risk (Table 3).

**Table 1:** Socio-demographic characteristics of the screened children

**Characteristics**	**Number**	**%**
**Sex**		
Male	173	52.4
Female	157	47.6
**Birth weight (kg)**		
Normal weight (2.5 kg-3.5)	251	76.0
Under weight (<2.5 kg)	26	7.9
Over weight (>3.5 kg)	53	16.1
**Duration of gestation**		
Term (37-40 weeks)	233	70.6
Pre-term (<37 weeks)	63	19.1
Post- term (>40 weeks)	34	10.3
**Method of delivery**		
SVD	162	49.7
C/S	63	19.1
Assisted	105	31.8

Key: *SVD* Spontaneous vertex delivery, *C/S* Cesarian section.

**Table 2:** Socio-demographic profile of the mothers

**Socio-demographics** **Maternal age at delivery (year)**	**Number**	**%**
15-19	9	2.7
20-24	61	18.5
25-29	123	37.3
30-34	84	25.5
35-39	40	12.1
40-44	11	3.3
45-49	2	0.6
**Maternal educational level**		
No formal education	12	3.7
Primary	47	14.2
JHS	133	40.3
SHS	80	24.2
Tertiary	58	17.6
**Parity of the mother**		
1	89	27.0
2	106	32.1
3	76	23.0
4	51	15.5
5	7	2.1
6	1	0.3

Key: *JHS* Junior High School, *SHS* Senior High School.

**Table 3:** Prevalence of developmental delay among the children

**Domains**	**Normal N(%)**	**At risk N(%)**	**Delayed N(%)**
Communication	281(85.1)	30(9.1)	19(5.8)
Gross motor domain	281(85.1)	27(8.2)	22(6.7)
Fine motor	255(77.3)	43(13.0)	32(9.7)
Problem solving	262(79.4)	35(10.6)	33(10.0)
Social/personal interraction	241(73.1)	48(14.5)	41(12.4)

Key: *N* Number.

### Associations between socio-demographic risk factors and developmental delay

Significant associations were found for the birth weight of the children and the duration of their gestation (χ^2^ = 13.4; p = 0.009 and χ^2^ = 10.5 ; p = 0.033, respectively) with the communication domain. Also, there were significant associations between the level of education attained by the mothers, duration of gestation of the children (χ^2^ = 16.3; p = 0.038 and χ^2^ = 10.6; p = 0.032 respectively), and the gross motor domain. The mode of delivery, parity and maternal age at delivery had no significant associations with the domains of the Ages and Stages Questionnaire. The results are presented in Table 4.

**Table 4:** Associations between socio-demographic factors and the domains of ages and stages questionnaires

**Risk factor**	**Communication**		**Gross motor**		**Fine motor**		**Problem solving**		**Social interaction**	
	***χ***^**2**^	**Sig.**	***χ***^**2**^	**Sig.**	***χ***^**2**^	**Sig.**	***χ***^**2**^	**Sig.**	***χ***^**2**^	**Sig.**
Birth weight of the children	13.4	Sig^*^	7.6	Insig	4.9	Insig	3.6	Insig	0.7	Insig
Duration of gestation of the baby	10.5	Sig^*^	10.6	Sig^*^	26.1	Insig	1.2	Insig	3.3	Insig
Mode of delivery	5.6	Insig	8.1	Insig	4.4	Insig	2.7	Insig	3.9	Insig
Maternal age at delivery	20.7	Insig	6.6	Insig	13.8	Insig	0.7	Insig	8.8	Insig
Maternal education	5.7	Insig	16.3	Sig^*^	9.2	Insig	11.9	Insig	8.1	Insig
Parity	18.3	Insig	5.4	Insig	9.3	Insig	9.5	Insig	3.9	Insig

Legend: Sig^*^ = Significant at p < 0.05; Insig = Insignificant.

## Discussion

The purpose of this study was to identify rural community dwelling children with DD at pre-school age and to determine its possible associations with selected socio-demographic risk factors. The results showed that 44.6% of the children screened had one form of DD or the other and the highest number (12.4%) was found in personal/social interaction. The least number (5.8%) was found to suffer delay in communication skills. These findings follow similar trend with the reports of a study in India where 46.8% of children were delayed in self-activity while 39.2% of them had delay in communication [18]. The difference in the proportions of the children found with delay, between the present and the previous studies may be attributed to several factors including evaluation tool utilized and environmental factors. Stages and Ages Questionnaires were used in the present study as screening tool while the Guide for Monitoring Child Development was adopted in the previous study. Also, a causal relation between environment and social interaction could be a crucial factor governing the level of social interaction of children on cultural and geogaphical context.

Our findings have corroborated the usual late identification of most defects and disabilities commonly found among children. This problem is caused by lack of periodic screening programmes in most rural welfare clinics in Ghana. The scenario is further aggravated by inadequate knowledge about birth defects among the nursing and the expectant mothers. Again, 273(82.7%) of the children screened were less than 12 months which is suggestive of common decline in the zeal of the mothers to visit welfare clinic with children older than 12 months of age. Most often, mothers whose children are within this age bracket assumed that their children are alright, thus may not warrant routine clinic attendance unless there is a major illness. Moreover, this study was conducted in a rural setting where level of education is low compared to sub-urban or urban communities. This finding has further buttressed the need to intensify awareness campaign about child health and development by all the stakeholders.

### Associations of the socio-demographic risk factors and developmental delay

The birth weight of the children and the duration of their gestation were significantly associated with communication skills in this study. Low birth weight has been shown to have large negative effects on mental development of which development of language skills forms a part [19,20]. Infants’ age at birth has been described as a strong predictor of neonatal health outcomes such as chances of survival, risk of medical complications, and timing for the achievement of development milestones [21]. Health risks associated with birth weight are however dependent on the weight categories. A substantial percentage of the children in this study had normal birth weight (76%) while 7.9% and 16.1% were underweight and overweight respectively. Low birth weight has been found to be associated with some complications including hypothermia, hypoglycemia, perinatal asphyxia, respiratory distress, anemia impaired nutrition, infection, neurological disorders and hearing defects [22].

More often than not, infants’ birth weights are normally considered along with gestational ages in determining neonatal health outcomes. Gill *et al*, had previously submitted that birth weight and gestational age in relation to birth weight categories could provide unique insights into infants’ birth history and developmental milestone achievement [20]. Our finding in this study therefore suggests that the birth weight status of the children and their gestational ages may have a link with the attainment of some developmental functions in pre-school aged children. The two factors specifically showed a link with communication skills in the present study.

Although, significant association was found between the gestational age and the communication skill in the present study, most authors were inconclusive about the effect of duration of gestation on DD. Communication disorders or preschool language delay is accompanied by a raft of problems. According to a study, the impacts of poor communication skills go well beyond early literacy development and ‘school readiness’ but could also extend to increasingly apparent associations with emotional, behavioural and social difficulties [23]. In addition, an emerging evidence indicates a very long-term sequelae that are not restricted solely to the school years or to children with serious clinical presentations [20].

Our study also indicates significant association between the duration of gestation and gross motor skills. This finding is in agreement with a similar study which concluded that preterm deliveries had a significant negative effect on motor and social development [24]. Most of the children (70.6%) in this study were delivered at term, 19.1% were delivered pre-term while 10.3% were delivered post-term. Infants with small for gestational age (<10th percentile) and those with large for gestational age (above 90th percentile) have been found to be accompanied by respiratory and motor dysfunctions respectively [25]. Late preterm infants born between 34 and 36 weeks gestation have recently been identified as having difficulty with motor functions in their preschool years [20]. Our study has also been able to establish a link between gestational age and gross motor skills.

Maternal educational level is also significantly associated with gross motor delay in this study. Majority of the mothers (40.3%) attained Junior High School level of education while 3.7% and 17.6% had no formal education and tertiary education respectively. A previous study has similarly reported that higher education among the parents had a positive effects on child development [26]. In the same vein, several studies have associated low parental educational levels and poverty with poor cognitive development of their children [27,28]. This outcome is not suprising given the rural nature of the participants’ environment which might have influenced their children’s delay in gross motor skills. The mode of delivery, parity and maternal age at delivery had no significant associations with the domains of Ages and Stages Questionnaire in this study. The finding about the mode of delivery is consistent with a previous study in which mode of delivery was found to cause no deleterious effects on child development [29]. On the contrary, Shaw et al reported that teen-age motherhood can be an important risk factor for poor childhood development [30]. Other authors however, opinned that maternal age alone is not a good index for predicting developmental outcomes in children [31].

### Limitations of the study

The main limitation in this study was the possible recall bias on the part of the mothers. This might have possibly arisen in the event of recalling the previous activities performed by a particular child in the event that the child could not perform the activity whilst none of the investigator was present. Although this forms part of the study protocols, the mother could forget some points. Also, the one-shot assessment of the children could also be a significant factor that could affect the outcome of the screening procedure. Future study to monitor the children’s development over a longer period is being planned to off set these limitations.

## Conclusion

Our findings demonstrate that an appreciable number of the screened children had various developmental delays. The personal/social interaction was the most prevalent developmental delay among the sampled children. Birth weight and gestational age of the children have possible link with communication skills while maternal educational level and gestational age provide an insight into a link with gross motor skills. Future study beyond pilot project on a wider scope will be necessary to co-opt other possible intervening factors.

## Competing interests

The authors declare that they have no competing interest.

## Authors’ contributions

AB participated in the project conceptualization, data entry, manuscript writing and data analysis, JQ assisted in data entry, manuscript writing and editing, LA, entered, coded and analyzed participants’ responses. All authors read and approved the final manuscript.

## Pre-publication history

The pre-publication history for this paper can be accessed here:

http://www.biomedcentral.com/1471-2431/13/119/prepub
